# Effects of Dietary Schizochytrium Algae as ω-3 PUFA Source on the Egg-Laying Quail Performance, Serum Indexes, and Egg Yolk Fatty Acids Contents

**DOI:** 10.3390/ani15010021

**Published:** 2024-12-25

**Authors:** Yifan Chang, Yue Xuan, Ruinan Zhang, Xuemei Ding, Qiufeng Zeng, Jianping Wang, Shiping Bai, Shanshan Li, Yan Liu, Yuchuan Chen, Keying Zhang

**Affiliations:** 1 Key Laboratory of Animal Disease-Resistance Nutrition, Ministry of Agriculture and Rural Affairs, Key Laboratory of Sichuan Province, Animal Nutrition Institute, Sichuan Agricultural University, Chengdu 611130, China; changyifan_980805@163.com (Y.C.); 71128@sicau.edu.cn (Y.X.); scu06zrn@foxmail.com (R.Z.); dingxuemei0306@163.com (X.D.); zqf@sicau.edu.cn (Q.Z.); wangjianping@sicau.edu.cn (J.W.); shipingbai@sicau.edu.cn (S.B.); 71449@sicau.edu.cn (S.L.); liuyan12334@163.com (Y.L.); 2 The Quail Science and Technology Backyard, Dongpo District, Meishan 620000, China; 18381092039@163.com

**Keywords:** quail, growth performance, ω-3 fatty acids

## Abstract

The objective of this experiment is to investigate the effects of SAP or SAO as ω-3 PUFA raw materials on production performance, egg quality, serum immunity, serum lipids, and fatty acid deposition patterns in the eggs of laying quails. Chinese yellow-feathered quails served as the experimental subjects. A single-factor design was employed to randomly assign 1288 quails into four treatment groups, with seven replicates per treatment and 46 birds in each replicate. The groups included a control group (basal diet with no SAP), 1.6% SAP, 3.2% SAP, and 0.8% SAP + 0.3% SAO. The results indicate that: (1) Compared to the control group, the 0.8% SAP + 0.3% SAO group exhibited a reduction in daily egg-laying rate and egg mass, alongside an increased FCR; (2) the 3.2% SAP group enhanced egg yolk color, while the 1.6% SAP group reduced eggshell thickness, and the 0.8% SAP + 0.3% SAO group increased eggshell thickness; (3) compared to the control group, the 3.2% SAP group decreased total cholesterol (TC) and high-density lipoprotein cholesterol (HDL-C) levels in serum; the addition of either the 3.2% SAP or the 0.8% SAP + 0.3% SAO group significantly elevated quail serum immunoglobulin M (IgM) levels (*p* < 0.05); (4) in comparison to the control group, the addition of SAP or with SAP increased the contents of monounsaturated fatty acids (MUFA), polyunsaturated fatty acids (PUFA), docosahexaenoic acid (DHA), and ω-3 PUFA in 56-day-old egg yolks while reducing the ω-6/ω-3 ratio (*p* < 0.05). These findings suggest that SAP as a source of ω-3 PUFA raw materials could improve quail health by improving lipid metabolism and immunity. 3.2% SAP was recommended as the optimal level to produce the enriched ω-3 PUFA quail eggs with the ω-3 PUFA ≥ 300 mg/100 g.

## Simple Summary

The current dietary structure of humans consists of a higher proportion of ω-6 and a lower proportion of ω-3 polyunsaturated fatty acids (ω-3 PUFAs). Given the limited availability of fish oil and other marine resources, which are also prone to oxidation, identifying stable sources of ω-3 PUFAs and their products has become a prominent research topic. This experiment investigates the effects of schizochytrium algae powder (SAP) or schizochytrium algae oil (SAO) as ω-3 PUFA raw materials on the production performance, egg quality, serum immunity, serum lipids, and fatty acid composition in the eggs of laying quails. The results indicate that the immune function and lipid metabolism of quails were improved with the addition of 3.2% SAP. This addition also significantly increases the ω-3 PUFA content in the eggs, providing a valuable reference for new sources of ω-3 PUFA in food.

## 1. Introduction

ω-3 PUFAs include the alpha-linolenic acid (ALA), eicosapentaenoic acid (EPA), and docosahexaenoic acid (DHA). Numerous studies have demonstrated that ω-3 PUFAs are beneficial for human brain development, neuronal processes, and retinal development, with a crucial role in the prevention of neurological diseases [1,2,3] and cardiovascular diseases by inhibiting inflammation and the body’s immune system [4,5,6]. Various governments and health organizations have recommended a dietary intake of ω-3 PUFAs ranging from 1.4 to 2.5 g per day, with EPA and DHA intake suggested to be between 140 and 600 mg per day. Current research indicates that the daily requirement for ω-3 PUFAs in adults is between 250 mg and 500 mg [7,8]. A study encompassing approximately 82% of the global population across 187 countries found that only 18.9% of individuals met the recommended daily intake of ω-3 PUFAs (250 mg/day) [9].

Since the human body lacks Δ-6-desaturase and is unable to synthesize fatty acids de novo [10], supplementing food with ω-3 PUFAs from external sources is an effective approach. Poultry eggs are particularly susceptible to dietary fatty acid regulation [11]. Research has demonstrated that supplementing diets with ω-3 PUFA raw materials, such as flaxseed, linseed oil, seaweed, and algae, can lead to the deposition of ω-3 PUFAs in poultry eggs and meat, facilitating effective deposition and transformation within the body [12,13,14,15]. In China, two agricultural industry standards, “ω-3 Polyunsaturated Fatty Acid Fortified Egg” (NY/T 4069-2021 [16], ω-3 PUFA ≥ 300 mg/100 g) and “Technical Specification for Production of ω-3 Polyunsaturated Fatty Acid Fortified Egg” (NY/T 4070-2021 [17]), were implemented in 2022. Due to quail’s characteristics, including a short incubation period (approximately 17–18 days), rapid growth, small size, high egg production, excellent reproductive performance, and low feeding costs [18], quail eggs are a popular food in China [19]. Some studies have indicated that incorporating ω-3 PUFA raw materials into the diets of quail, such as flaxseed, linseed oil, rapeseed oil, sunflower seed oil, and shark liver oil, can enhance the ω-3 PUFA content in quail eggs [20,21,22]. SAP contains a high ω-3 PUFAs level (18%) with DHA, and SAO contains more DHA than SAP [23]. However, there is currently a paucity of research focused on the production of ω-3 PUFA-enriched quail eggs using SAP or SAO.

This experiment investigated the effects of SAP or SAO on the production performance, egg quality, serum immunity and lipids, and fatty acid deposition patterns in quail eggs. The findings will support the scientific and rational utilization of SAP or SAO as feed ingredients for the production of quail eggs enriched with ω-3 PUFA. Additionally, this research provides theoretical insights for developing food sources that are rich in ω-3 PUFA.

## 2. Materials and Methods

This study was approved by the Animal Care and Use Committee, Sichuan Agricultural University (Ethic Approval Code: SICAUAC202110-2; Chengdu, China).

### 2.1. Trial Design, Diets, and Quail Management

The trial was conducted at Quail Science and Technology Backyard in Dongpo District, Meishan, China. The Chinese yellow-feathered quails were procured from the Yunge Quail Professional Cooperative located in Dongpo District, Meishan, China. A single-factor design was employed to randomly assign 1288 quails (12 weeks old) into four treatments, with seven replicates per treatment and 46 birds per replicate. The treatments included the CON diet (basal diet) with no SAP, 1.6% SAP, 3.2% SAP, and 0.8% SAP + 0.3% SAO. The basal diet was formulated according to the nutrient recommendation of the National Research Council (NRC) (1994) for quail. The SAP (Fat: 41.90%, DHA: 22.50%) and SAO (Fat: 97.50%, DHA: 52.50%) were obtained from the commercial company and substituted the lard in the basal diet based on the fat content (Table 1). The level of SAP was used based on the content of DHA and the relationship of dietary ω-3 PUFAs level with the content of ω-3 PUFAs in chicken eggs [24]. SAO contains more DHA and was used with SAP, which may increase the DHA content in eggs.

The quails were raised in multi-layer cages with 46 quails per cage and free access to diet in mash and water. Manure was manually cleaned every 3–4 days. The room temperature was maintained between 25 and 30 °C with 16 h lighting.

### 2.2. Sample Collection

The quail egg yolk samples were collected on the 56th day of the trial. Two quails from each replicate were randomly selected. Blood was collected from the jugular vein after weighing and centrifuged at 3000 r/min for 10 min to obtain serum, which was stored at −20 °C for subsequent determination of serum lipid metabolism indicators. The quails were euthanized by bleeding from the jugular vein, and the abdominal cavity was promptly opened to extract the complete liver, which was weighed and divided into three parts (left, middle, and right), bagged, and stored at −20 °C for liver fatty acid measurement.

### 2.3. Productive Performance

Egg-related data, including the total number of eggs laid, egg weight, and number of dead quail, were recorded daily. The feed consumption for each replicate was also recorded on a weekly basis. Then, the following indexes were calculated: laying rate (%), egg weight (g), average daily feed intake (ADFI), feed conversion ratio (FCR), egg mass, and mortality. The laying rate is calculated as the total number of eggs produced divided by the cumulative number of birds per day multiplied by 100. The egg mass is calculated as the total egg weight divided by the total days and the cumulative number of birds per day. Egg weight is calculated as the total egg weight divided by the total number of eggs. Average daily feed intake is obtained by the total feed consumption divided by the daily cumulative number of birds. The feed conversion ratio (FCR) is calculated by the total feed consumption divided by the total egg weight. The mortality is calculated as the number of dead quails during the trial divided by the number of birds at the beginning of each phase.

### 2.4. Egg Quality

On the 56th day of the experiment, six eggs were randomly collected from each replication, and the egg quality was assessed within 24 h. The measured indicators included egg weight, yolk color, Haugh unit, eggshell thickness, egg shape index, eggshell strength, and the calculation of the eggshell ratio and yolk ratio. The Japanese Robotmation egg quality analyzer EMT-5200 (The general agent of Japan robotmation egg quality inspection system in China is Nanjing Yaoen Instrument Equipment Co., Ltd., Nanjing, China), purchased from Sichuan Agricultural University, was utilized to measure egg weight, albumen height, yolk color, and Haugh unit. Eggshell strength was evaluated using an eggshell strength tester, while eggshell thickness was measured with a micrometer. Yolk weight and eggshell weight were determined using electronic scales. The longitudinal diameter, transverse diameter, and yolk height were measured using vernier calipers.

### 2.5. Serum Biochemistry

The contents of total cholesterol (TC), triglycerides (TG), high-density lipoprotein cholesterol (HDL-C), and low-density lipoprotein cholesterol (LDL-C) in the serum and liver of 20-week-old quails were measured using a fully automatic blood biochemistry analyzer from Sichuan Agricultural University. An ELISA kit from the Nanjing Jiancheng Institute of Biology was employed for these determinations. Additionally, immune parameters in quail serum, including immunoglobulin G (IgG) and immunoglobulin M (IgM), were assessed using an ELISA kit from Jiangsu Enzyme Immunization Industrial Co., Ltd. in Nanjing, China.

### 2.6. Egg Yolk Fatty Acid Composition

On the 56th day of the experiment, six eggs were randomly collected from each repetition, and the yolks were separated and weighed. After thoroughly mixing, the yolks were placed in a plastic bag. The yolks were then freeze-dried at −80 °C and subsequently weighed. The samples underwent extraction and saponification. Following methyl esterification and extraction, they were placed into vials for testing. According to the method as described [25]. The analysis was conducted using a Shimadzu 2010 Plus gas chromatograph (Shimadzu, Kyoto, Japan, GC-2010 PLUS) from Sichuan Agricultural University, with fatty acid composition expressed as a percentage of total fatty acids.

### 2.7. Data Analysis

Data analysis was conducted using SAS 9.4 software. The production performance, fresh egg quality, egg yolk fatty acid content, as well as lipid metabolism-related factors, were analyzed through one-way ANOVA analysis in the SAS program. Tukey’s method was employed for multiple comparisons.

## 3. Results

### 3.1. Productive Performance

As shown in Table 2, the 1.6% SAP or 3.2% SAP groups showed laying rate and egg mass at 1–4 weeks compared to the control group. There was no significant difference (*p* > 0.05), but the 0.8% SAP + 0.3% SAO group is significantly lower than that of the other groups. However, this trend did not last at 5–8 w, but there was a near significant difference for the laying rate (*p* = 0.058) and egg mass (*p* = 0.071) at 1–8 weeks. There was no significant difference among treatments for ADFI, egg weight, or mortality at each phase, but there was a significant difference for FCR at 1–4 weeks or 1–8 weeks, with the highest for the 0.8% SAP + 0.3% SAO group than that of the other groups.

### 3.2. Egg Quality

As shown in Table 3, compared to the control group, the egg yolk color in the 3.2% SAP group showed a significant increase. Additionally, the eggshell thickness in the 0.8% SAP + 0.3% SAO group also significantly increased, while the eggshell thickness in the 1.6% SAP group significantly decreased (*p* > 0.05).

### 3.3. Serum Indexes

As shown in Table 4, compared to the control group, the levels of TC, TG, and HDL-C in the 3.2% SAP were significantly different (*p* < 0.05), while no significant difference was observed in LDL-C and TG. However, there was a decreasing trend in LDL-C levels in the 3.2% SAP group (*p* = 0.065).

Compared to the control group, serum IgM levels were significantly elevated in the groups receiving 3.2% SAP and 0.8% SAP combined with 0.3% SAO (*p* < 0.05).

### 3.4. Egg Yolk Fatty Acid Composition

The effects of two sources of ω-3 PUFA raw materials on the fatty acid composition and content of quail egg yolk after 56 days are presented in Table 5. The use of 1.6% SAP, 3.2% SAP, or 0.8% SAP with 0.3% SAO did not significantly influence the contents of saturated fatty acids (SFA) or unsaturated fatty acids (UFA) in yolk but significantly increased the polyunsaturation in the egg yolk fatty acid (PUFA) content. Additionally, it significantly reduced the monounsaturated fatty acids (MUFA) in egg yolks. Then, significant differences existed in the content of ω-3 and ω-6 fatty acids or ω-6/ω-3 in yolk among the different treatment groups (*p* < 0.05). The use of 1.6% or 3.2% SAP or 0.8% SAP with 0.3% SAO significantly increased the content of DHA and ω-3 PUFA in the yolk or ω-3 PUFA in quail eggs with a dose–response (*p* < 0.05).

## 4. Discussion

Wu et al. and Bruneel et al. have found that incorporating 1%, 2%, 4%, 5%, 8%, or 10% schizochytrium powder into the diet does not adversely affect the production performance of laying hens. This suggests that adding between 0% and 10% of this powder will not negatively impact laying hens’ performance [26,27]. In contrast, Shibani observed that adding more than 3% fish oil to the diet resulted in a decrease in the egg production rate of laying hens [28]. Additionally, Elkin et al. reported that including 4% algae oil in the diet significantly reduced the egg production rate and increased the feed-to-egg ratio [29]. Santana’s findings indicated that the addition of 1%, 2%, 3%, and 4% schizochytrium powder to the diet did not affect the production performance of Japanese quail [30]. Our results also showed that the use of 1.6% or 3.2% SAP in the quail diet did not significantly influence the quail egg production rate, feed intake, FCR, average egg weight, or egg mass. However, compared to the control or 1.6% SAP or 3.2% SAP, the addition of 0.8% SAP with 0.3% SAO significantly decreased the egg production rate and egg mass at 1–4 w (*p* < 0.05), with a significant increase in FCR at 1–4 w and 1–8 w, while there was a decreasing trend for the laying rate (*p* = 0.058) and egg mass (*p* = 0.071) at the 1–8 w period. These negative results may be due to the addition of SAO.

The color of egg yolk serves as a crucial sensory evaluation index for assessing egg quality. This coloration is primarily influenced by fat-soluble pigments present in the diet, such as carotenoids and lutein. Typically, various pigments in the feed undergo esterification during digestion, after which they are decomposed into a free state. These pigments then combine with fatty acids and are absorbed into the body. Once absorbed, the pigments are concentrated in the egg yolk after passing through the ovary [31]. Numerous studies have demonstrated that the inclusion of ω-3 polyunsaturated fatty acids (ω-3 PUFA) in the diet significantly enhances the yolk color of both chicken and quail eggs [31,32,33,34]. One study indicated that incorporating 1%, 2%, 3%, and 4% Schizochytrium algae powder into the diet resulted in a proportional increase in the color intensity of quail egg yolks [30]. Furthermore, this study revealed that a diet containing 3.2% algae powder led to a higher yolk color in the group at 8 weeks compared to the control group, corroborating previous research findings.

The eggshell serves as a protective barrier that prevents microorganisms from infiltrating the egg. Eggs with inferior shells are more vulnerable to contamination. Increased eggshell thickness and strength reduce the likelihood of damage to commercial eggs during storage and transportation. In this study, the group receiving 0.8% algae powder combined with 0.3% algae oil exhibited a significant increase in the eggshell thickness of quail eggs. Furthermore, incorporating 0.9% dietary spirulina powder into quail feed has been shown to enhance eggshell quality. Neijat et al. reported that the addition of 0.2%, 0.4%, and 0.6% DHA algal oil did not adversely affect eggshell quality [35]. This finding aligns with previous research in poultry. The observed increase in eggshell thickness may result from the inclusion of schizochytrium oil, which enhanced the metabolism of calcium, phosphorus, and other essential minerals. However, it was observed that the thickness of eggshells in the 1.6% algae powder group was reduced, which contradicts the findings of Abd El-Hack [34]. This discrepancy may be attributed to poor intestinal absorption of calcium and phosphorus in the animals of this experimental group, which could inhibit the transmission of calcium ions and disrupt the formation of the shell membrane, ultimately leading to alterations in the eggshell structure and a decrease in eggshell thickness [36,37].

Serum lipid content serves as an indicator of lipid metabolism in animals. In poultry, the primary site for the production of total cholesterol (TC) and triglycerides (TG) is the liver. Compared to the control group, the 3.2% algae powder group exhibited significant increases in TC and high-density lipoprotein cholesterol (HDL-C), while no significant differences were observed in low-density lipoprotein cholesterol (LDL-C) and TG levels. Notably, there was a decreasing trend in LDL-C levels in the 3.2% algae powder group (*p* = 0.065). Under normal conditions, the endogenous triglycerides (TG) in egg-laying poultry are synthesized in the liver and subsequently transported outside the body through low-density lipoprotein cholesterol (LDL-C). Meanwhile, HDL-C facilitates the transfer of TG from peripheral tissues to the liver, where it is processed through the bile pathway before being excreted from the body [38]. Several studies have demonstrated that incorporating 1%, 1.5%, and 2% spirulina combination products, or adding 0%, 0.25%, 0.5%, and 1% Salina to the diet, can significantly reduce total cholesterol (TC) and triglycerides (TG) in quail serum [34,39]. Consistent with the findings of this article, the precise mechanism underlying the reduction in TC remains unclear. In mammals, current research suggests that docosahexaenoic acid (DHA) inhibits diacylglycerol O-acyltransferase 2 (DGAT2) in the liver, thereby increasing plasma lipase activity, which reduces hepatic fat production and promotes beta-oxidation of fatty acids in the liver [40,41]. Avellone et al. reported that the decrease in TG levels in the blood is attributable to the inhibitory effect of ω-3 polyunsaturated fatty acids (ω-3 PUFA) on the key enzyme responsible for TG synthesis in the liver [42].

In this trial, LDL-C exhibited a decreasing trend, while HDL-C decreased significantly. Previous studies have indicated that HDL-C levels typically increased when serum LDL-C decreased [43], which is inconsistent with the findings of this study. Bujo et al. demonstrated that the lipid metabolism of laying hens prior to egg laying involves the transport of substantial amounts of lipoproteins and micronutrients into oocytes, thereby maintaining somatic cell homeostasis [44]. During the peak laying period, a considerable volume of lipid transport is necessary for the transfer of lipoproteins to the ovary, which is essential for yolk formation; LDL and HDL are critical factors in sustaining lipid balance. Consequently, the observed results may be attributed to the unique characteristics of lipid transport in poultry during the peak egg-laying period. Furthermore, the aforementioned studies suggest that schizochytrium algae powder enhances the capacity for cholesterol transport from the bloodstream to the liver, indicating that the addition of algae powder could positively influence liver function and lipid metabolism in quails.

The content of immune factors in animals can reflect their health status. In birds, IgA, IgY (IgG), and IgM are the primary antibodies [45]. IgM is the first type of antibody produced during the activation of the body’s humoral immunity. It accumulates in the mucosal immune system and plays a crucial role in the pathogenic process [46]. IgM exhibits both antiviral and antibacterial properties [47]. IgG is primarily involved in secondary immunity. This experiment demonstrated that adding 3.2% algae powder to the diet significantly increased serum IgM levels. Alghamdi et al.’s research found that a dosage of 1–4 mL/kg of spirulina significantly elevated the serum IgM content in quails [48]. Similarly, Abdel-Wahab et al. reported that incorporating 3% spirulina powder into the diet significantly enhanced serum IgM and IgG levels in quails [49], which aligns with the findings of the IgM presented in this article. It is speculated that ω-3 PUFA promotes the binding of IgM to antigens and initiates the complement cascade reaction, facilitating the phagocytosis of pathogens by macrophages [50]. However, this experiment did not find the concentration of IgG to be statistically significant. It is possible that an immune response has recently occurred. The specific reasons for this finding require further investigation. The results indicate that incorporating ω-3 PUFA sources, such as algae powder, into the diet can enhance the humoral immune function of the animal body, thereby improving its immune response capabilities against viruses and bacteria.

Feng et al. reported that incorporating 1.25%, 2.5%, and 5% microalgae oil or fish oil into the diet resulted in a significant increase in the content of DHA and total ω-3 PUFA in eggs [51]. Wu et al. [26] added 1%, 2%, 4%, and 8% microalgae powder to the diets of laying hens with the result of a significant increase in the ω-3 polyunsaturated fatty acids (PUFA) in eggs and a notable reduction in the ω-6/ω-3 ratio. It has been reported in the literature that at a dietary dose of 8% microalgae powder, ω-3 content exhibited the highest PUFA deposition [26]. Similarly, Satana et al. [30] observed comparable results when incorporating 1–4% schizochytrium powder into quail diets, further supporting the notion that the inclusion of ω-3 PUFA in poultry diets can markedly enhance the ω-3 PUFA content in egg yolks, consistent with the findings of this study. Additionally, some research has indicated that docosahexaenoic acid (DHA) in egg yolk is predominantly found in the yolk’s phospholipids. When DHA or PUFA is involved in the formation of egg yolk glycerophospholipids, it tends to preferentially bind to the more stable Sn-2 position. This binding is influenced by competition between ω-3 and ω-6 fatty acids, resulting in a significant decrease in ω-6 PUFA and a substantial increase in ω-3 PUFA [52,53,54].

Our results demonstrate that SAP is a good ω-3 PUFA-enriched ingredient to significantly enhance the contents of PUFA, DHA, and ω-3 PUFA in quail egg yolk with no significant influence on the contents of SFA and UFA while also markedly decreasing the ω-6/ω-3 ratio. These PUFA, DHA, and ω-3 PUFA enrichment effects were dose–response with the dietary SAP increased. According to the requirement of the ω-3 PUFA-enriched egg, the content of ω-3 PUFA in the egg should be 300 mg/100 g egg (NY/T 4069-2021). The ω-3 PUFA (mg/100 g egg) of quail egg produced by the use of 3.2% SAP was 389.98 mg/100 g and could achieve the requirement of NY/T 4069-2021 [16] for a chicken egg with a higher yolk color value. The content of DHA in the diet of 0.8% SAP with 0.3% SAO was similar to that of the 1.6% SAP diet, and also the DHA content in quail egg yolk was similar among the two treatments. It shows that SAO is also an efficient ω-3 PUFA source for DHA-enriched quail egg production. However, the level of SAO in the diet may be needed more, which may result in a further decrease in laying performance.

## 5. Conclusions

Through the experiments conducted, we found that incorporating a specific dose of ω-3 PUFA source material into the diet can enhance the ω-3 PUFA content in quail egg yolks. By comparing the groups supplemented with SAP alone at 1.6%, 3.2%, or 0.8% with SAO 0.3%, we observed no negative differences in production performance between the two groups with SAP. However, the production performance of the treatment group receiving SAP 0.8% with SAO 0.3% was adversely affected, leading us to conclude that the 0.3% SAO is not the most suitable source. Despite this, the combination of 0.8% SAP and 0.3% SAO also yielded similar ω-3 PUFA contents in quail egg yolk. Conversely, the diet supplemented with 3.2% SAP demonstrated no negative impact on production performance with improved immune function and lipid metabolism while significantly increasing the n-3 PUFA content in egg yolks.

Therefore, the SAP is a good ω-3 PUFA source to produce the quail egg with the ω-3 PUFA enrichment, especially the DHA-enriched quail egg. The suitable dietary level is 3.2% to produce the ω-3 PUFA-enriched quail egg with the ω-3 PUFA ≥ 300 mg/100 g.

## Figures and Tables

**Table 1:** Composition and nutrient level of diets (as-fed basis).

Ingredients	CON	1.6% SAP	3.2% SAP	0.8% SAP + 0.3% SAO
Corn	50.93	50.53	50.13	51.95
Soybean meal	29.00	28.75	28.50	29.60
Lard	4.10	3.65	3.20	3.40
Corn gluten meal (60%)	4.00	4.00	4.00	4.00
Alcohol grains	3.00	2.50	2.00	1.00
Schizochytrium algae oil	0.00	0.00	0.00	0.3
Schizochytrium powder	0.00	1.60	3.20	0.80
Limestone	2.00	2.00	2.00	2.00
Calcium carbonate	4.80	4.80	4.80	4.80
Dicalcium phosphate	1.00	1.00	1.00	1.00
NaCl	0.30	0.30	0.30	0.30
Mineral premix ^1^	0.05	0.05	0.05	0.05
Vitamins premix ^2^	0.02	0.02	0.02	0.02
L-Lysine HCl	0.33	0.33	0.33	0.31
DL-Methionine	0.26	0.26	0.26	0.26
L-threonine	0.03	0.03	0.03	0.03
Choline chloride (50%)	0.15	0.15	0.15	0.15
Phytase	0.03	0.03	0.03	0.03
Total	100	100	100	100
Calculated levels				
ME (kcal/kg)	2.907	2.905	2.903	2.906
Crude protein%	20.08	20.08	20.08	20.08
Calcium%	2.90	2.90	2.90	2.90
Total phosphorus%	0.53	0.55	0.57	0.53
Available Phosphorus%	0.30	0.30	0.30	0.30
DHA%	0.00	0.36	0.72	0.34
Lysine%	1.09	1.09	1.09	1.09
Methionine%	0.55	0.55	0.55	0.55
Threonine%	0.68	0.68	0.68	0.68
Tryptophan%	0.19	0.19	0.19	0.19

^1^. Provided per kilogram of diet: Vitamin A 9900 IU, Vitamin D_3_ 2610 IU, Vitamin E 15.3 IU, Vitamin K_3_ 1.8 mg, Vitamin B_1_ 1.62 mg, Vitamin B_2_ 4.86 mg, Vitamin B_6_ 2.7 mg, Vitamin B_12_ 0.013 mg, Niacin 18.9 mg, Calcium Pantothenate 7.56 mg, Biotin 0.81 mg, and Folic Acid 0.036 mg. ^2^. Provided per kilogram of diet: Copper (CuSO_4_·5H_2_O) 6.5 mg, Iron (FeSO_4_·H_2_O) 70 mg, Manganese (MnSO_4_·H_2_O) 60 mg, Zinc (ZnSO_4_·H_2_O) 60 mg, Iodine (KI) 0.40 mg, and Selenium (Na_2_SeO_3_) 0.40 mg.

**Table 2:** Effects of dietary ω-3 PUFA addition on quail production performance.

Items	CON	1.6% SAP	3.2% SAP	0.8% SAP + 0.3% SAO	SEM	*p*-Value
Laying rate (%)						
1–4 w	62.51 ^a^	56.60 ^a^	59.99 ^a^	43.22 ^b^	3.623	0.004
5–8 w	70.85	69.17	68.56	61.22	4.667	0.488
1–8 w	66.68	62.89	64.28	52.22	3.780	0.058
ADFI (g)						
1–4 w	18.68	18.02	18.57	17.63	0.551	0.509
5–8 w	20.13	19.23	20.76	20.40	0.853	0.628
1–8 w	19.40	18.63	19.66	19.02	0.676	0.719
FCR						
1–4 w	3.05 ^b^	3.23 ^b^	3.00 ^b^	4.56 ^a^	0.297	0.003
5–8 w	2.66	2.67	2.83	3.19	0.158	0.083
1–8 w	2.85 ^b^	2.94 ^b^	2.92 ^b^	3.87 ^a^	0.209	0.006
Egg weight (g)						
1–4 w	10.06	10.25	10.47	10.12	0.142	0.218
5–8 w	10.82	10.80	11.17	10.88	0.138	0.234
1–8 w	10.44	10.53	10.82	10.50	0.120	0.148
Egg mass (g/quail/d)						
1–4 w	6.30 ^a^	5.80 ^a^	6.28 ^a^	4.38 ^b^	0.404	0.008
5–8 w	7.68	7.48	7.63	6.64	0.514	0.456
1–8 w	6.99	6.64	6.96	5.51	0.427	0.071
Mortality (%)						
1–4 w	0.77	0.83	0.67	0.65	0.247	0.949
5–8 w	1.21	0.73	0.84	2.11	0.367	0.056
1–8 w	0.99	0.78	0.76	1.38	0.211	0.309

Abbreviations: ADFI, average daily feed intake; FCR, feed conversion ratio. a, b Means with different superscripts within the same line differ significantly (*p* ≤ 0.05).

**Table 3:** Effects of dietary ω-3 PUFA addition on quail egg quality.

Items	CON	1.6% SAP	3.2% SAP	0.8% SAP + 0.3% SAO	SEM	*p*-Value
Egg shape index	0.77	0.79	0.78	0.78	0.0004	0.140
Egg weight (g)	11.44	11.27	11.49	11.48	0.316	0.958
Eggshell strength, kg/cm^3^	1.13	1.04	1.12	1.08	0.042	0.389
Haugh unit	75.65	78.32	78.14	78.19	0.999	0.203
Yolk color	4.52 ^b^	4.84 ^ab^	5.27 ^a^	4.43 ^b^	0.206	0.037
Egg yolk ratio%	31.52	31.92	32.40	31.50	0.552	0.630
Eggshell ratio%	9.59	9.55	9.71	9.46	0.147	0.667
Eggshell-thickness/μm	234.13 ^b^	212.84 ^c^	235.96 ^ab^	251.36 ^a^	5.563	0.001

a, b, c Means with different superscripts within the same line differ significantly (*p* ≤ 0.05).

**Table 4:** Effects of dietary ω-3 PUFA supplementation on serum lipids of quails.

Items	CON	1.6% SAP	3.2% SAP	0.8% SAP + 0.3% SAO	SEM	*p*-Value
HDL-C (μmol/g)	1.56 ^a^	1.34 ^ab^	0.90 ^b^	1.51 ^a^	0.154	0.022
LDL-C (μmol/g)	1.73	1.17	0.77	1.14	0.238	0.065
TC (μmol/g)	4.24 ^a^	3.5 ^ab^	2.51 ^b^	3.63 ^ab^	0.391	0.035
TG (μmol/g)	10.42 ^a^	6.31 ^ab^	5.88 ^b^	7.26 ^ab^	1.131	0.038
IgM (g/L)	13.94 ^b^	15.65 ^ab^	17.37 ^a^	17.74 ^a^	0.916	0.033
IgG (g/L)	69.41	70.02	64.50	65.73	2.262	0.612

TC, total cholesterol; HDL-C, high-density lipoprotein cholesterol; TG, triacylglycerol; LDL-C, low-density lipoprotein cholesterol; IgG, immunoglobulin G; IgM, immunoglobulin M. a, b Means with different superscripts within the same line differ significantly (*p* ≤ 0.05).

**Table 5:** Effects of dietary ω-3 PUFA supplementation on fatty acid composition of quail egg yolks.

Items	CON	1.6% SAP	3.2% SAP	0.8% SAP + 0.3% SAO	SEM	*p*-Value
SFA	36.67	37.51	36.78	37.88	0.441	0.178
UFA	63.33	62.49	63.21	62.11	0.441	0.178
MUFA	50.33 ^a^	48.08 ^b^	47.77 ^b^	47.73 ^b^	0.444	0.0026
PUFA	12.33 ^b^	14.34 ^a^	15.20 ^a^	14.31 ^a^	0.335	<0.0001
ω-6 PUFA	11.57 ^a^	10.76 ^b^	9.85 ^c^	10.48 ^bc^	0.254	0.0018
ω-3 PUFA	1.11 ^c^	3.58 ^b^	4.95 ^a^	3.82 ^b^	0.121	<0.0001
ω-6/ω-3	11.79 ^a^	3.01 ^b^	1.98 ^b^	2.74 ^b^	0.552	<0.0001
DHA (mg/100 g egg yolk)	181.14 ^c^	1137.71 ^b^	1359.59 ^a^	1165.21 ^ab^	83.961	<0.0001
ω-3 PUFA (mg/100 g egg yolk)	280.19 ^c^	1243.79 ^b^	1491.13 ^a^	1274.67 ^ab^	89.470	<0.0001
ω-3 PUFA (mg/100 g egg)	59.31 ^c^	265.74 ^b^	389.98 ^a^	263.56 ^b^	283.13	<0.0001

Levels were calculated as SFA: C6:0 + C10:0 + C12:0 + C14:0 + C15:0 + C16:0 + C17:0 + C18:0 + C20:0 + C21:0 + C22:0 + C23:0 + C24:0; MUFA: C16:1n-7 + C18:1n-9 + C20:1n-9 + C22:1n-9 + C24:n-9; ω-6 PUFA: C18:2n-6 + C18:3n-6 + C20:3n-6; ω-3 PUFA: C18:3n-3 + C20:5n-3 + C22:6n-3. a, b, c Means with different superscripts within the same line differ significantly (*p* ≤ 0.05).

## Data Availability

Data are available upon request from the corresponding authors.
